# Testicular Damage following Testicular Sperm Retrieval: A Ram Model Study

**DOI:** 10.1155/2017/2472805

**Published:** 2017-09-27

**Authors:** Jens Fedder, Niels Marcussen, Maja D. K. Fedder, Birte Engvad

**Affiliations:** ^1^Centre of Andrology, Odense University Hospital, Odense, Denmark; ^2^Fertility Clinic, Odense University Hospital, Odense, Denmark; ^3^Department of Pathology, Odense University Hospital, Odense, Denmark

## Abstract

The aim of this study was to evaluate the possible development of histological abnormalities such as fibrosis and microcalcifications after sperm retrieval in a ram model. Fourteen testicles in nine rams were exposed to open biopsy, multiple TESAs, or TESE, and the remaining four testicles were left unoperated on as controls. Three months after sperm retrieval, the testicles were removed, fixed, and cut into 1/2 cm thick slices and systematically put onto a glass plate exposing macroscopic abnormalities. Tissue from abnormal areas was cut into 3 *μ*m sections and stained for histological evaluation. Pathological abnormalities were observed in testicles exposed to sperm retrieval (≥11 of 14) compared to 0 of 4 control testicles. Testicular damage was found independently of the kind of intervention used. Therefore, cryopreservation of excess sperm should be considered while retrieving sperm.

## 1. Introduction

Since the early nineties, large and increasing numbers of infertile couples have had Intracytoplasmic Sperm Injection (ICSI) using sperm retrieved from the testis or epididymis. In many azoospermic men, particularly men with obstructive azoospermia (OA), testicular sperm retrieval is performed as a randomly located open biopsy using a scalpel [1, 2]; testicular sperm aspiration (TESA) using a needle, gauge 19–26 [3, 4]; or testicular sperm extraction (TESE) using a TruCut “gun” needle, gauge 14 or 18 [5, 6]. In some cases with nonobstructive azoospermia or cryptozoospermia, opaque seminiferous tubules may be isolated by open surgery using loop glasses (magnification ×4) [7] or microdissection TESE (mTESE; magnification ×25) [8] in order to minimize vascular injury and the amount of testis tissue removed. In some men with Klinefelter's syndrome (KS), unilateral subcapsular orchiectomy may be used as an alternative to micro-TESE [9].

Several studies have shown that children born after ICSI using testicular or epididymal sperm are healthy and the risk of malformations, except from hypospadias and cryptorchidism, is not increased [10, 11]. In men with OA, sperm retrieval rates are close to 100% even after repeated procedures [12, 13]. However, when TESE was performed at an earlier procedure in men undergoing repeated sperm retrieval, the pregnancy rate was only 27% (although sperm were found in all cases) compared to a 48% pregnancy rate where epididymal sperm were retrieved and used the previous time [12], suggesting that operations on the testis may impair sperm function.

An association between testicular fibrosis and reduced semen quality has been found in cattle [14]. Studies using rat models suggest that multiple needle biopsies cause larger irreversible scar tissue formations than TruCut biopsies do [15]. However, rats and other rodents might be less representative of humans, since they have smaller testicles, which furthermore, by the use of the cremaster muscles, are drawn into the abdomen during the winter time. Another weakness of the aforementioned study is that the latest follow-up was done as early as four weeks after testis retrieval [15].

Hematomas and scar tissue are also found in human testicles after biopsy [16]. Using ultrasonography, focal lesions have been detected in 4 out of 61 testicles (6.6%) from men with OA or nonobstructive azoospermia (NOA) 3 months after TESA using a 19-gauge butterfly needle. However, 9 months later, it was not possible to refind the lesions [17]. In a minor study where most participants were diagnosed with OA, none showed abnormalities by testicular ultrasonography 3 months after TESE using a 14-gauge TruCut needle [18].

Pain does not necessarily reflect the magnitude of scar formation. Nevertheless, it is worth mentioning the study of Westlander et al. [17] who reported intense discomfort experienced by 4 out of 35 (11%) azoospermic men undergoing TESA using a 19-gauge needle. Additionally, Wood et al. [19] found a higher postoperative pain level in 63 men undergoing TESA as open biopsy compared to 22 men undergoing percutaneous epididymal sperm aspiration (PESA) two weeks after sperm retrieval attempts using a Visual Analog Scale (VAS). However, the pain perception was significantly less than pain perception after previous vasectomy and vasectomy reversal [19].

With this study, we wanted to investigate whether sperm retrieval leads to chronic testicular changes and which sperm retrieval technique is the better choice. Since it is not possible to examine the human testicles histologically after operation, we have looked for an animal model. Compared to humans, most animals have a larger testis weight/body weight ratio [20], and not many other species than ruminants have their testicles permanently present in a scrotum outside the body to the same extent as humans. Therefore, we performed prospective, experimental studies in a ram model.

## 2. Materials and Methods

**Figure 1:**
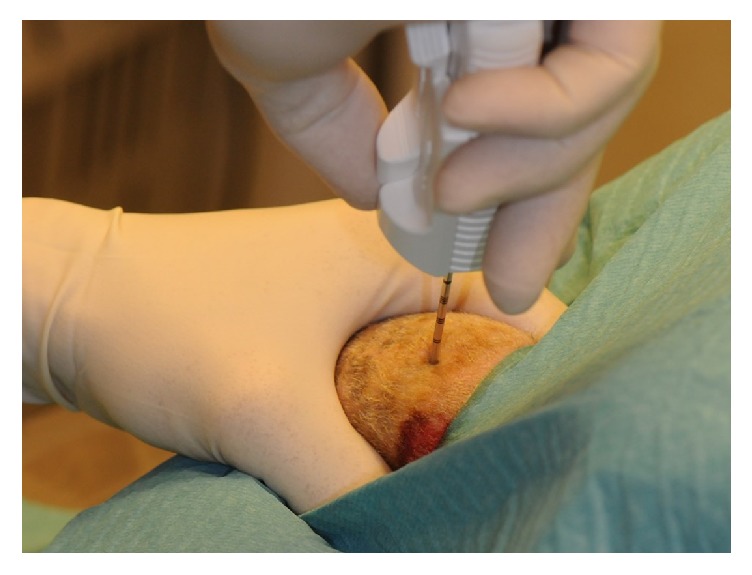
After razing and disinfection with chlorhexidine digluconate 0.5% in ethanol 96% and staining with curcumin, TESE was performed using a 14-gauge TruCut needle, which was introduced percutaneously through the scrotal skin and into the ram testis.

The experiments were done on Shropshire rams, 6–8 months of age, originating from Tyrevoldsdal, Skanderborg, Denmark. Each testicle was exposed to a given surgical procedure (Figure 1) or left unoperated on as control. The rams were given Xylazinum, 8 mg (~0.4 mL; Rompun Vet., Bayer AG, Leverkusen, Germany) as premedication 10 min before operation. When the animals were relaxed, Propolipid 20 mg (~2 mL; propofol, Fresenius Kabi AB, Uppsala, Sweden) was given intravenously through an 18-gauge angiocatheter (BD Venflon Pro, Becton Dickinson, Helsingborg, Sweden) into the foreleg and supplemented when the occasion requires to retain universal anesthesia.

**Figure 2:**
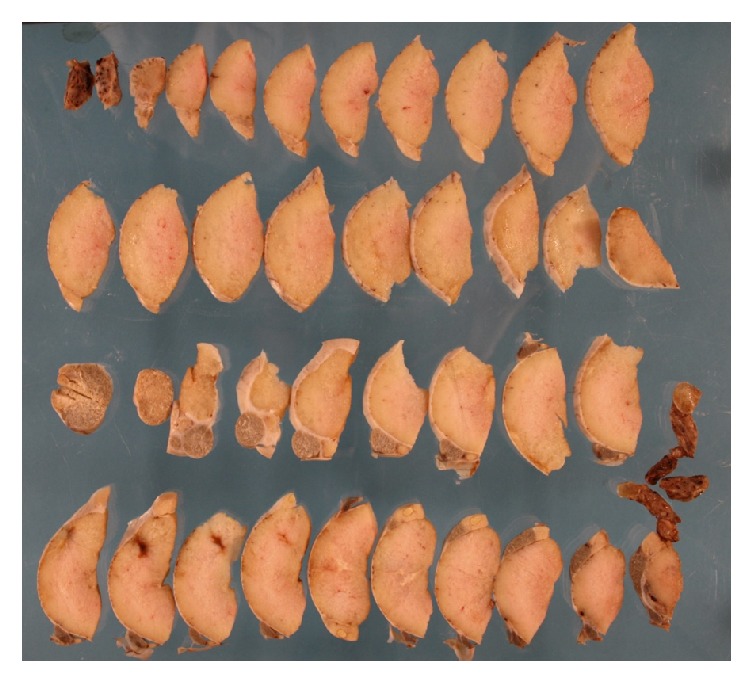
A ram testis, which had undergone multiple biopsies with a 19-gauge needle (TESA) cut into slices, which have been placed systematically on a glass plate. Areas with hematoma or scar tissue can be identified macroscopically.

Three months later, the animals were sacrificed, and the testicles and epididymides adhering to the testicles were removed for histological examination. Immediately after removal, each testis was cut longitudinally from upper to lower pole 2/3 of the diameter from the side opposite to the epididymis. This allows the formaldehyde (4%) to penetrate the testis tissue. The formalin fixed tissue was afterwards completely cut into slices of about 1/2 cm thickness and systematically put on a glass plate, making it possible to identify and localize macroscopic abnormalities, for example, areas with extensive fibrosis (Figure 2). Tissue was cut from the abnormal areas and embedded in paraffin, and then the tissue was cut into 3 *μ*m sections and stained with hematoxylin-eosin and Masson trichrome for histological evaluation.

### 2.1. Experiment 1

Three rams underwent sperm retrieval performed as TESE with TruCut needle (14G; Angiotech Medical Device Technologies Inc., Florida, USA (from April 2013: Aragon Medical Devices Inc., Texas, USA)) in two testicles or as multiple biopsies (TESA) with a 19G needle in another two testicles. The remaining two testicles were left unoperated on as control. Each procedure was performed in one right and one left testicle. Thus, the two testicles in each animal were in every case treated in two different ways.

### 2.2. Experiment 2

In three of six rams, one testicle underwent traditional open biopsy, where a small testis sample quelling out through a small incision in the* tunica albuginea* was cut using small scissors. The* tunica albuginea* and* tunica vaginalis* were closed with running Vicryl suture, while the skin was sutured with three Vicryl knots. TruCut biopsies (14G) were performed in seven testicles, while the remaining two testicles served as control. In four of the seven testicles undergoing TruCut biopsy in Experiment 2, the needle was inserted centrally near the* rete testis*. In the other three cases, the needle was inserted in the testicular periphery (distant from the* rete testis*). Identical procedures were never performed on each side of a given ram, and we always avoided hitting the* mediastinum testis* since it contains vessels. Damage to these might cause bleeding and intratesticular hematoma.

### 2.3. Approval

The study was approved in 2012 as method testing by the Animal Experiments Inspectorate (approval 2010/561-1949).

## 3. Results

**Figure 3:**
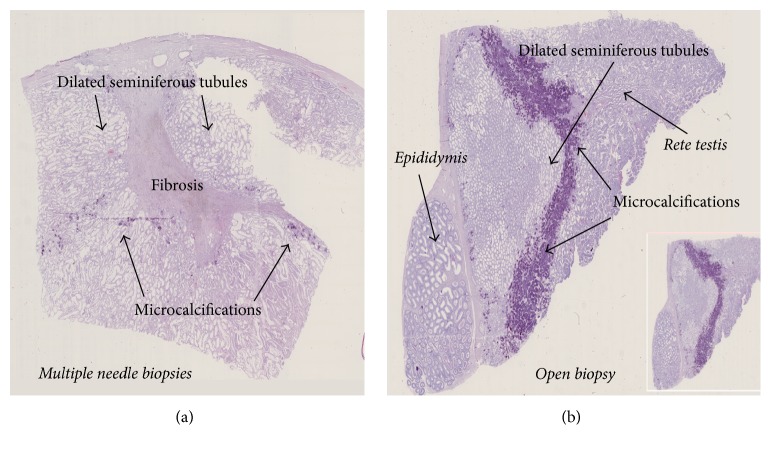
Microscopic section of testicular tissue. (a) A 30 × 8 mm area with fibrosis after multiple needle biopsies with a 19-gauge needle. The hyalinized area is surrounded by areas with dilated and atrophic seminiferous tubules and with microcalcifications (which are the reasons for the folding artefacts of the preparation). (b) After a traditional open biopsy, a huge area with a lot of microcalcifications and surrounded by dilated and atrophic seminiferous tubules is observed near the* rete testis*.

Most testicles exposed to any sperm retrieval procedures showed areas with microcalcifications (11 of 14) and fibrosis (9 of 14) surrounded by areas with dilated seminiferous tubules (Figures 3(a) and 3(b)). In comparison hereto neither microcalcifications nor fibrosis could be detected in the 4 control testes that were unoperated on (Table 1).

The first experiment left the impression that TruCut biopsies located near the* rete testis* caused larger damage than TruCut biopsies located in the testicular periphery. Therefore, we compared the morphological damage seen after peripheral testicular TruCut biopsies and central TruCut biopsies located near the* rete testis.*

In the second experiment, three rams had TruCut biopsies taken in the testicular periphery and four near the* rete testis*. Independently of biopsy location, areas with fibrosis and calcifications were seen. The size of the affected area varied, and the extent could not be associated with the location of the biopsy. In biopsies taken near the* rete testis*, the fibrotic process did not expand in the peripheral direction of the seminiferous tubules. In no case was the* rete testis* damaged. If the affected tissue has a length of 30 mm and a radius of 4 mm as shown in Figure 3(a), the destroyed tissue has a volume of 0.25 mL (≈3.0 × (0.4)^2^ × *π*/6).

Since the caput of the epididymis in rams prolonged around the upper pole of the testis, the epididymis was in a few cases hit during sperm retrieval from peripheral or central areas of the testis using the TruCut needle. This caused development of a sperm granuloma in a couple of cases.

## 4. Discussion

This experimental ram study shows that pathological, testicular abnormalities, such as scar tissue and microcalcifications, were found very often after conventional testicular sperm retrieval, whether traditional open biopsy, multiple needle biopsy (TESA), or TruCut biopsy (TESE) was performed. The pathological testicular abnormalities were observed when TruCut biopsies were taken near the* rete testis* as well as in the testicular periphery far away from the* rete testis*. Fibrosis and microcalcifications were not seen in control testicles not exposed to sperm retrieval or other operative procedures. Such pathological changes in the testicles may impede sperm production and testicular function relatively more in small testicles or testicles with sperm production in only small foci.

The strength of using an animal model is that it is possible to remove and systematically evaluate the whole testis by, for example, histological examination at systematically chosen time intervals following the operative procedure. Furthermore, the strength of using a ruminant species such as the sheep is that the testicles are permanently present in a scrotum outside the body similar to humans. However, a weakness using an animal model is that almost all animal species have much higher testis weight/body weight ratio compared to humans [20]. A damaged area of a given size may be relatively more serious in a testicle with a small volume. Therefore, since almost all animals have excellent sperm production, it is not easy to develop an animal model for evaluation of the influence of sperm retrieval on testicles in NOA with production of sperm in only small foci. Since tissue for microscopy was taken from areas showing macroscopic abnormalities, areas which might show only microscopic changes as a result of the procedure are missed using the present study design. Finally, it is difficult to measure pain or discomfort in an animal model.

Several techniques have been used for testicular sperm sampling. For testicular fine needle aspirations (FNA, TEFNA, and TESA), needles or butterfly needles with sizes 19G [1, 21], 21G [1, 22, 23], or 23G [3, 24] are used. Finally, Arïdoğan et al. [25] used a 26G needle only allowing cytological evaluation. Mercan et al. [23] replaced the abbreviation FNA with PTSA (percutaneous testicular sperm aspiration) to underline that the procedure was performed without an incision in the scrotal skin.

TruCut biopsies with 14G [5, 6, 26] allow histological examination of the sampled material. Although TruCut biopsies are performed with needles of larger sizes, the procedure may be less traumatic than FNA [18], in which multiple in-and-out movements in all directions at every testicular puncture are often done [3].

Vanderzwalmen et al. [27] found sperm by TESE in 97.4% (112/115) of men with obstructive azoospermia, in whom sperm were detected by diagnostic testicular biopsy previously. Conversely, it was only possible to refind testicular sperm in 69.4% (95/137) of men with nonobstructive azoospermia treated the same way. These results are in accordance with Fedder et al. [12], who found sperm in 100% of 76 repeated sperm retrieval procedures (TESA, PESA, and TESE) in men with obstructive azoospermia, and with Hussein [28], who found a decreased chance of finding sperm by repeat sperm retrieval in men with hypospermatogenesis and maturation arrest.

The risks of impairment of the blood supply and of postsurgical fibrosis seem to be increased by multiple testicular tissue sampling, and testicular abnormalities detected by ultrasonography remain up to 6 months after surgery [16]. In patients with NOA, Schlegel and Su [16] could retrieve testicular sperm in only 1 of 4 men by a repeated TESE performed 2–4 months after the initial procedure compared to 80% of 15 men by a repeated TESE performed 6 months after the initial procedure. Such scanty data have guided colleagues to recommend at least 6 months of convalescence between repeating testicular sperm sampling procedures in men with NOA.

From these data, it is suggested that OA and NOA should be treated in different ways, and omission of diagnostic TESE should be considered in NOA. An alternative to diagnostic TESE in men with NOA might be an examination for the presence of ejaculated spermatids. If spermatids—but no mature sperm—are detected in the ejaculate, the diagnosis is NOA. However, when spermatids are not ejaculated, it is not possible to distinguish between lack of production of testicular germ cells (e.g., SCO syndrome) and OA [2, 29].

The possible risk of development of anti-sperm antibodies (ASA) after sperm retrieval procedures is not fully clarified. Steele et al. [18] detected ASA of the IgG class in serum samples from 4 of 20 azoospermic men 4 weeks after sperm retrieval using a 14-gauge TruCut (gun) needle. They suggested the cause to be previous vasectomy in 2 cases and cystic fibrosis (CFTR) mutations and congenital bilateral absence of the* vas deferens *(CBAVD) in the other 2 men [18]. However, serum was not examined for the presence of ASA before sperm retrieval, which is a weakness of the study. In another study, two of 35 men (5.7%) were positive for IgG ASA preoperatively, and the IgG titers were maintained 3 and 6 months after multiple TESAs using a 19-gauge butterfly needle. Furthermore, 3 men developed borderline levels (~not significantly raised) of ASA after the TESAs [17].

In some men with very impaired spermatogenesis, a few sperm are present in the ejaculates now and then. This condition is termed cryptozoospermia [30]. In practice, at least two ejaculates without sperm are required for the diagnosis: azoospermia. The raw ejaculates as well as pellets after centrifugation are routinely examined for the presence of sperm [31].

The risk of postsurgical fibrosis found in humans [16] was confirmed and semiquantified in this ram study. Although the length of the interval between repeated sperm retrieval procedures is less important for OA patients with regard to finding sperm due to far more universal spermatogenesis [12], areas inside the testis may still be injured due to impairment of the blood supply. This increases the risk of development of calcification, fibrosis, and antisperm autoimmunity.

Studies in humans have shown that testosterone levels decrease after TESE [32, 33] as well as after micro-TESE [34, 35]. The androgen deficiency is to some extent reversible within 12 months depending on the etiology and histological diagnosis [34, 35]. Repeated or multiple testicular biopsies may increase the risk of androgen deficiency [28, 32].

In conclusion, testicular damage can be found independently of the kind of intervention used. For patients with OA, we therefore recommend considering retrieval of epididymal spermatozoa, given that epididymal reconstructive surgery is excluded as an option. In any case, we recommend cryopreservation of excess sperm obtained by testicular sperm retrieval to minimize the risk of further sperm retrieval.

Further research is needed focusing on additive effects of repeated sperm retrieval procedures, including the length of the intervals between repeated procedures. Since the acute changes seen at ultrasonography in retrospective studies have been found to be reduced after mTESE compared to conventional TESE [36, 37], it could be interesting to also evaluate mTESE in the ram model. In these studies, it is also relevant to evaluate short- and long-term changes in levels of testosterone, FSH, and LH after the respective procedures performed once or several times.

## Figures and Tables

**Table 1:** Pathological findings in the ram testicles removed 3 months after a sperm retrieval procedure.

Procedure	Areas with fibrosis	Microcalcifications	Tubular atrophy/dilation
Open biopsy	2 of 3	2 of 3	2 of 3
Multiple needle biopsies	2 of 2	2 of 2	2 of 2
TruCut biopsy	5 of 9	7 of 9	9 of 9
Control	0 of 4	0 of 4	0 of 4
